# Photocatalytic Hydrogen Evolution from Artificial Seawater Splitting over Amorphous Carbon Nitride: Optimization and Process Parameters Study via Response Surface Modeling

**DOI:** 10.3390/ma15144894

**Published:** 2022-07-14

**Authors:** Michell K. T. Chee, Boon-Junn Ng, Yi-Hao Chew, Wei Sea Chang, Siang-Piao Chai

**Affiliations:** 1 Multidisciplinary Platform of Advanced Engineering, Chemical Engineering Discipline, School of Engineering, Monash University, Jalan Lagoon Selatan, Bandar Sunway 47500, Selangor, Malaysia; michell.chee@monash.edu (M.K.T.C.); ng.boon.junn@monash.edu (B.-J.N.); yi.chew@monash.edu (Y.-H.C.); 2 Mechanical Engineering Discipline, School of Engineering, Monash University, Jalan Lagoon Selatan, Bandar Sunway 47500, Selangor, Malaysia; chang.wei.sea@monash.edu

**Keywords:** photocatalysis, amorphous carbon nitride, hydrogen evolution, Box–Behnken design, process study, seawater splitting

## Abstract

Photocatalytic water splitting has garnered tremendous attention for its capability to produce clean and renewable H_2_ fuel from inexhaustible solar energy. Until now, most research has focused on scarce pure water as the source of H_2_, which is not consistent with the concept of sustainable energy. Hence, the importance of photocatalytic splitting of abundant seawater in alleviating the issue of pure water shortages. However, seawater contains a wide variety of ionic components which have unknown effects on photocatalytic H_2_ production. This work investigates photocatalytic seawater splitting conditions using environmentally friendly amorphous carbon nitride (ACN) as the photocatalyst. The individual effects of catalyst loading (X_1_), sacrificial reagent concentration (X_2_), salinity (X_3_), and their interactive effects were studied via the Box–Behnken design in response surface modeling towards the H_2_ evolution reaction (HER) from photocatalytic artificial seawater splitting. A second-order polynomial regression model is predicted from experimental data where the variance analysis of the regressions shows that the linear term (X_1_, X_2_), the two-way interaction term X_1_X_2_, and all the quadratic terms (X_12_, X_22_, X_23_) pose significant effects towards the response of the HER rate. Numerical optimization suggests that the highest HER rate is 7.16 µmol/h, achievable by dosing 2.55 g/L of ACN in 45.06 g sea salt/L aqueous solution containing 17.46 vol% of triethanolamine. Based on the outcome of our findings, an apparent effect of salt ions on the adsorption behavior of the photocatalyst in seawater splitting with a sacrificial reagent has been postulated.

## 1. Introduction

Hydrogen (H_2_) is one of the best potential clean fuels in terms of its environmental friendliness and high-energy density. H_2_ provides clean burning and generates energy up to 142 kJ/g under standard conditions, which is significantly higher than any conventional fuel in the current energy pool [1]. Several technologies are available to produce H_2_, such as natural gas reforming, methanol reforming, and water splitting. Among them, photocatalytic water splitting, the conversion of pure water driven by solar energy into usable chemical energy (i.e., H_2_), has garnered significant attention [2,3,4,5,6,7,8,9,10,11,12]. However, pure water is scarce, which contradicts the purpose of deriving high-availability fuels. 

Seawater is an inexhaustible water resource, attributed to its extensive geographic coverage on Earth (i.e., about 97 percent of water reserves on the Earth are saline) [13]. Seawater has an average salinity of 3.5% (35 g/L) globally, where the dissolved salts are composed predominantly of sodium (Na^+^) and chloride (Cl^-^) ions [14]. The high availability of seawater offers tremendous benefits for the application of photocatalytic H_2_ evolution reaction (HER), especially for areas where pure water is not accessible. However, the presence of a large quantity of dissolved ionic components in seawater has cut both ways. For example, Cl^-^ ions might pose a competitive reaction with oxidation sites that suppress a sacrificial reagent’s adsorption [15]. However, a low concentration of Na^+^ could also promote the adsorption of sacrificial reagents [16]. The mechanism of seawater splitting is still in the exploratory stage. Therefore, research into seawater splitting explores the potential of inorganic ions to facilitate HER and surmount the contemporary water scarcity issue [17,18,19,20,21]. 

To achieve this feat, research into polymeric graphitic carbon nitride (GCN) in energy application has attracted tremendous attention attributed to its appealing properties: (1) facile preparation method from inexpensive precursors such as urea and melamine, (2) high chemical stability, and (3) visible light activity [22]. However, the photocatalytic performance of GCN is bound by the limitation of severe charge recombination and moderate light absorption range [23]. Amorphous carbon nitride (ACN), a derivative of GCN, can harness an even wider range of solar energy to split H_2_O into H_2_ molecules. ACN can be synthesized by breaking the intramolecular structure of GCN via a simple thermal treatment [24]. The withdrawal of inter-heptazine units from the graphitic structure of GCN can induce the shaping of an amorphous phase and extends the light absorption. Furthermore, the formation of mid-gap states associated with defects can facilitate the electron hole separation, leading to enhanced photocatalytic performance. More importantly, ACN is environmentally benign and chemically stable, complying with the criteria for sustainable energy production. Our previous publication has demonstrated the better performance of ACN in photocatalytic H_2_ production over GCN [24]. Thus, ACN was employed as the model photocatalyst in this study. As the effects of process parameters on the HER activity in photocatalytic seawater splitting are still not well explored, in the present work, we have investigated the three main process parameters, i.e., catalyst loading, sacrificial reagent concentration, and salinity, through R.S.M. Their individual and interactive effects towards the HER performance were reported.

## 2. Materials and Methods

### 2.1. Materials

Melamine (99.0%, Sigma Aldrich, St. Louis, MI, USA), hexachloroplatinic (IV) acid hydrate (≥99.9%, Sigma Aldrich, St. Louis, MI, USA), triethanolamine (≥99.0%, Sigma Aldrich, Darmstadt, Germany), and ethanol (Grade AR 96%, Fisher Scientific, Shah Alam, Malaysia) were used as obtained without further purification. Deionized (DI) water was used throughout the experiment. 

### 2.2. Photocatalysts Synthesis 

The pristine GCN was synthesized from a typical thermal condensation process. In brief, melamine (2 g) was placed in alumina crucibles covered with lids and calcined at 500 °C for 4 h in a muffle furnace, and the ramping rate was set to 5 °C min^−1^. After cooling naturally to room temperature, the solid was collected, ground into finer powder, and washed with DI water several times. ACN was synthesized via a post-annealing treatment of GCN under an inert environment. Typically, pristine GCN (0.3 g) was subjected to calcination at 620 °C for 2 h at a heating rate of 5 °C min^−1^ in a tube furnace under a continuous N_2_ flow (10 mL min^−1^) to generate an inert environment. Prior to the calcination process, the air inside the tube furnace was evacuated by purging with N_2_ gas at a high flow rate for 30 min. The details of these experimental procedures have been reported in our previous studies [24].

### 2.3. Samples Characterisation

The crystallographic properties of the samples were obtained from X-ray diffraction (XRD) measurements using an X-ray diffractometer (Bruker D8 Discover) equipped with Ni-filtered Cu Kα radiation (λ = 0.15406 nm). The scan rate was 0.02° s^−1^, the accelerating voltage was 50 kV, and the current was 40 mA. The optical absorption spectra of the samples were acquired via an ultraviolet-visible (U.V.-Vis) spectrometer (Agilent Cary 100), and BaSO_4_ was used as a reflectance standard. The optical band gap of the samples was further acquired from a Tauc plot, [h*v*.F(R)]^1/n^ vs. h*v*, where n = ½ for the indirect band gap. The surface microstructure was acquired from a field emission scanning electron microscope (FESEM) (Hitachi SU8010). The surface area of the samples was measured using the Micromeritics ASAP 2020. 

### 2.4. Electrochemical Measurements

The flat band potentials of the samples were acquired from a Mott–Schottky plot using a C.H.I. 6005E electrochemical workstation (CH Instruments, Austin, TX, USA) equipped with a three-electrode photoelectrochemical setup. The three electrodes are a platinum rod (counter electrode), Ag/AgCl saturated with 3 M KCl (reference electrode), and a working electrode. The working electrode was prepared by drop-casting a fixed amount of photocatalyst in ethanol suspension onto a 1 × 1 cm^2^ fluoride-doped tin oxide (FTO) glass slide. The analysis was conducted using 0.5 M Na_2_SO_4_ as the electrolyte, and the working electrode was illuminated by a Xenon arc lamp (CHF-XM-500 W, 500 W) equipped with an AM 1.5 filter. 

### 2.5. Preparation of Artificial Seawater and Photocatalytic H_2_ Evolution Measurements

The artificial seawater environment was simulated by dissolving the sea salt (Aqua Ocean Reed Plus Marine Salt, Qingdao Sea Salt Aquarium Technology) in deionized water (DI water) with a composition as shown in Table S1. To prepare artificial seawater with a salinity of x_3_ g/L, x_3_ g of ocean sea salt was dissolved in 1 L of DI water. In a typical photocatalytic HER experiment, 30 mg of the photocatalyst was dispersed in a 40 mL aqueous solution containing triethanolamine (TEOA), hexachloroplatinic (IV) acid hydrate (3 wt% Pt), and sea salts. The reaction mixture was purged with N_2_ at a high flow rate for 30 min to evacuate the air from the reaction vessel. The HER was carried out by subjecting the reaction mixture to the illumination of simulated sunlight generated from a Xenon arc lamp (CHF-XM-500W, 500 W). An AM 1.5 filter was used to simulate the solar irradiance spectrum. The reaction mixture was stirred and purged continuously with N_2_ gas at 5 mL min^−1^ for the entire reaction under atmospheric conditions. The output gas from the vessel was analyzed simultaneously at every 0.5 h interval by the Agilent 7820A online gas chromatography (G.C.) instrument equipped with a thermal conductivity detector. 

### 2.6. Experimental Design and Analytical Methods

The effect of 3 factors, i.e., catalyst loading (X_1_), TEOA concentration (X_2_), and salinity (X_3_) on HER rate (Y), were evaluated via Design-Expert software (version 13) by employing the R.S.M. with the Box–Behnken design. Three design levels (+1, 0, −1) were used for each factor, as shown in Table 1. 

The best model, second-order polynomial regression, was selected via the Whitcomb score (a heuristic scoring system) and has the general equation as shown below:

(1) $$Y=A+\overset{k}{\underset{i=1}{\sum }}B_{i}x_{i}+\overset{k}{\underset{i=1}{\sum }}C_{i}x_{i}{}^{2}+\overset{k}{\underset{1\leq i<j}{\sum }}D_{i}x_{i}x_{j}$$

where *Y* is the predicted response, *A* is the constant term, *x_i_* and *x_j_* are the studied variables, *B_i_* is the effect of the linear term, *C_i_* is the effect of the squared term, *D_i_* is the effect of interaction between variable *i* and *j*, and *k* is the total variables.

## 3. Results and Discussion

### 3.1. Structural and Optical Properties of Amorphous Carbon Nitride

**Figure 1:**
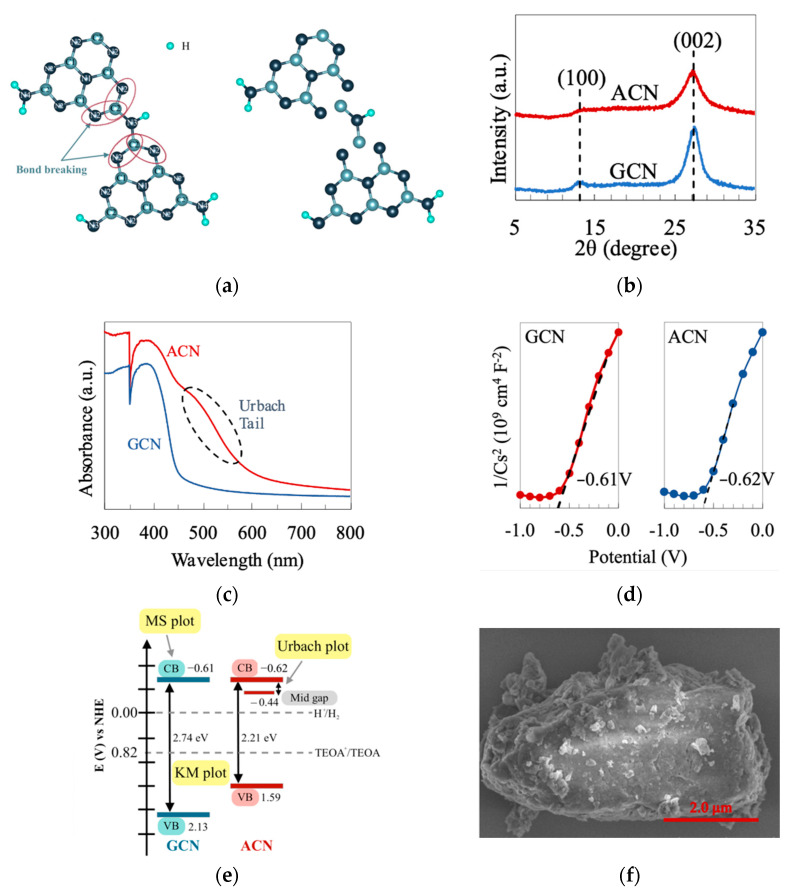
(**a**) Molecular structure of the repeating unit for GCN and ACN. (**b**) XRD patterns; (**c**) UV-Vis absorption spectra; (**d**) Mott–Schottky plots and (**e**) band structure diagram for GCN and ACN. (**f**) FESEM image of ACN.

ACN is a derivative of polymeric GCN, which has a defected molecular structure, as shown in Figure 1a. A small CNHC group is removed from the junction between heptazine units to form an amorphous structure. Firstly, XRD characterized the phase structures of ACN and GCN. As delineated in Figure 1b, both samples exhibit a similar XRD pattern with two apparent peaks at ca. 13.1° and 27.2°. The peak at 13.1° can be indexed as a (100) plane of g-C_3_N_4_, corresponding to the tri-*s*-triazine units. On the other hand, the typical (002) peak at 27.2° refers to the periodic interplanar stacking aromatic system of g-C_3_N_4_ [25]. Careful observation shows that the XRD peaks of ACN (FWHM of peak at (002) = 2.176) are slightly broader than that of GCN (FWHM of the peak at (002) = 1.794). This implies a higher degree of amorphousness of ACN with a more loosely packed structure than GCN [26]. To provide a discerning understanding of the optical properties of g-C_3_N_4_ after amorphization, the samples were examined using UV-Vis. As shown in Figure 1c, ACN renders a more red-shifted absorption edge than GCN. The defected molecular structure causes the ACN to have a relatively lower band gap (2.21 eV) than GCN (2.72 eV). Besides, there is an absorption tail in the UV-Vis profile of ACN within the visible region. The presence of this absorption tail (also known as the Urbach tail) is ascribed to the band trailing effect correlated to the formation of the mid-gap energy level. The energy of the mid-gap level can be calculated via the linearized form of the Urbach equation, as shown in Equation (2) [27]. Furthermore, the flat band potential of ACN and GCN were determined to be −0.62 and −0.61 eV using the Mott–Schottky measurement (Figure 1d). Working in conjunction with UV-Vis data, the samples’ plausible band structures are shown in Figure 1e. The defected structure introduces a new energy level between the conduction band (C.B.) and valence band (V.B.). This newly formed energy level, also known as the mid-gap level, provides an electron-trapping effect and suppresses charge recombination, as evident by the P.L. spectra in Figure S1. Furthermore, ACN displays a 3D structure inherited from GCN, as shown in the FESEM image (Figure 1f and Figure S2). The B.E.T. surface area of ACN was examined (S_ACN_ = 46.61 m^2^/g) and depicted a significantly higher value than that of GCN (S_GCN_ = 26.57 m^2^/g). The photocatalytic performance of GCN and ACN is presented in Figure S3, where the ACN shows a more superior activity than GCN in both pure water and seawater with TEOA as the sacrificial reagent.

(2) $$\mathrm{Linearized}\;\mathrm{Urbach}\;\mathrm{equation}:\;\ln\;\alpha =(\frac{1}{E_{u}})\;hv+\ln\alpha_{0}$$

where α is the absorption coefficient, h*v* is the photon energy, and E_u_ is the Urbach energy (the energy difference between C.B. and mid-gap state).

### 3.2. Modeling Fitting and Validation

In this design, 15 experimental runs conducted at different factorial levels of the three factors in Table 1 were planned. Analysis of variance (ANOVA) was employed to analyze the influences of the three factors—catalyst loading (X_1_), TEOA concentration (X_2_), and salinity (X_3_) on the response—HER rate, Y. The predicted and adjusted R^2^ values by ANOVA in Table 2 were used to select the best model for the computational study. The best model—second-order polynomial regression was chosen for its highest Scores 1 and 2 to predict the HER rate, Y. The response Y—HER rate is related to the input factors via the following equation (in terms of coded factor):

Rate (µmol/h) = 6.09 + 0.9045 X_1_ + 1.77 X_2_ + 0.6295 X_3_ + 2.16 X_1_X_2_ + 0.6283 X_1_X_3_ + 0.0127 X_2_X_3_ − 2.03 X_1_^2^ − 2.16 X_2_^2^ − 1.51 X_3_^2^ (3)

**Figure 2:**
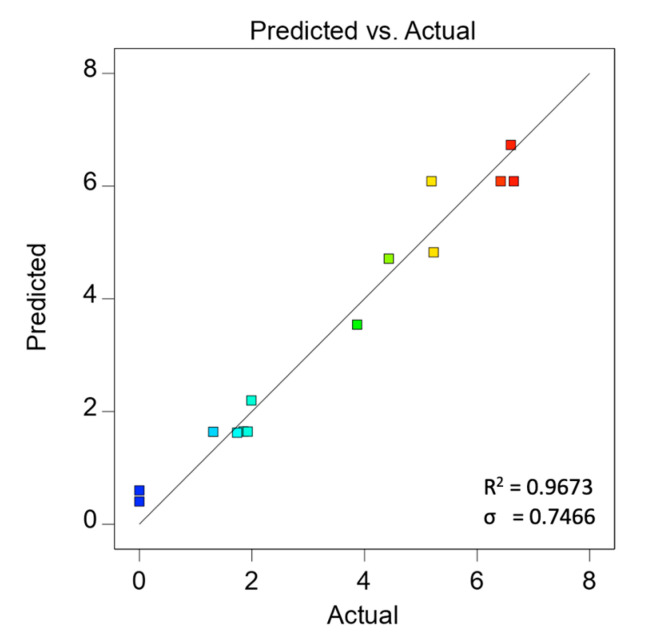
Predicted versus actual values for HER rate based on second-order polynomial regression.

The predicted HER rates calculated based on Equation (3) and the HER rate obtained experimentally are tabulated in Table 3. The graph of experimental data versus predicted data from Table 3 was plotted in Figure 2, showing a logical correlation between the predicted and actual values of the HER rate. Moreover, the R^2^ with a value close to 1 and a low root mean square estimation, σ, suggests the well-fitting of experimental data with the selected model. 

### 3.3. Terms in Model Equation

Table 4 shows variance analysis results by ANOVA. The *p*-value is the coefficient by ANOVA to evaluate the significance of the source towards the response in the second polynomial regression, where a *p*-value of less than 0.05 is statistically tested as significant. As the polynomial regression for HER rate is of second order, hence, there are three types of terms in general, i.e., the first order (X_1_, X_2_, X_3_), the two-way interaction (X_1_X_2_, X_1_X_3_, X_2_X_3_) and lastly, the pure quadratic (X_1_^2^, X_2_^2^, X_3_^2^). The analytical results in Table 4 show that the linear terms X_1_ and X_2_ are significant and have *p*-values of 0.0187 and 0.0013, respectively. The linear term X_3_ is also relevant but is less critical as it had a *p*-value of 0.0628. X_1_X_2_ is the only two-way interaction term that tested significant (*p*-value = 0.0025). This also indicates that the interaction between catalyst loading and TEOA concentration is vital in enhancing the HER rate. The pure quadratic terms X_1_^2^, X_2_^2^, and X_3_^2^ are also significant terms in Equation (3). The significance of these terms can be observed from their greater constant value in the equation.

### 3.4. Effect of Factors towards HER from Seawater Splitting

**Figure 3:**
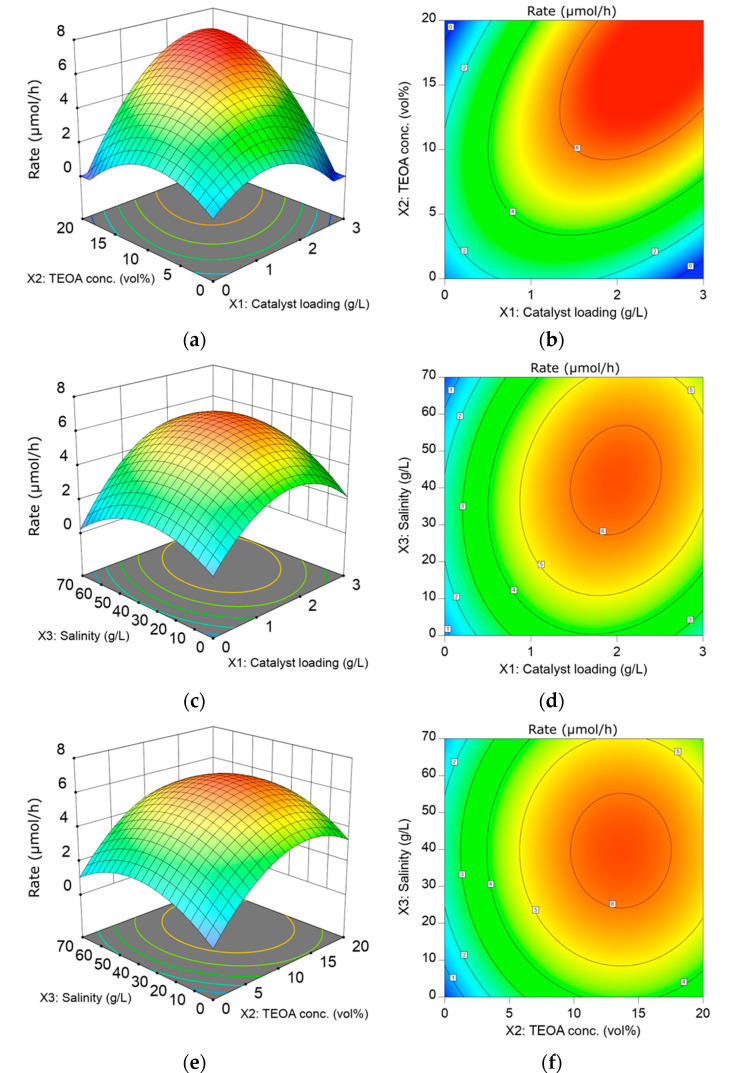
The 3D response surface plots and contour plots show the interaction between (**a**,**b**) catalyst loading and TEOA concentration; (**c**,**d**) catalyst loading and salinity; (**e**,**f**) TEOA concentration and salinity based on the HER rate with the controlled factor keep at level 0.

The effect of ACN loading, TEOA concentration, and salinity on the average reaction rate over a 3 h time duration is depicted in the 3D surface and contour plots (Figure 3). Figure 3a,c shows the effect of catalyst loading on the HER rate at different TEOA concentrations and salinity, respectively. As observed in Figure 3a, the HER rate increases from 0 to the highest, 6.86 µmol/h, when catalyst loading increases from 0 to c.a. 2.67 g/L in a solution containing 20 vol% TEOA in artificial seawater (33 g_sea salt_/L). The positive effect of increasing catalyst loading towards HER rate is also observed in Figure 3c from 0 to c.a. 1.93 g/L catalyst loading, but deterioration in HER rate occurs thereafter. The improvement in photocatalytic activity could be attributed to the increase in available surface-active sites and the total amount of available surface charge. The HER rate decreases at any greater catalyst loading (X_2_ > 1.93 g/L) regardless of the greater available surface site. Intuitively, the deterioration in the HER activity is due to the screening of light by the excessive catalyst. This occurs when the concentration of the photocatalyst reaches the light exposure threshold of the reactor. 

Figure 3a,e is of interest to reveal the effect of TEOA concentration on HER activity. In Figure 3a, the HER rate increases with increasing TEOA concentration, and the highest HER rate of 6.85 µmol/h is observed at the upper boundary of the designated TEOA concentration (20 vol%). The improvement in HER rate is due to the enhanced adsorption rate of TEOA onto the photocatalyst surface in higher TEOA concentrations. In Figure 3e, the highest observed HER rate occurs at c.a. 14 vol% TEOA concentration, reaching a value of 6.52 µmol/h. Resembling the effect of catalyst loading, the HER rate shows deterioration after reaching the optimum. The further deterioration could be related to the competition between the two reactants (TEOA and water) for the adsorption site on the photocatalyst surface. The increase in the total volume of TEOA results in the limited surface coverage by water on photocatalyst, which prompts the predominance of TEOA in adsorption activity. Thus, the HER rate deteriorates when TEOA concentration exceeds its optimum.

The contour plot in Figure 3b shows the interaction between catalyst loading and TEOA concentration. It is observed that the optimum TEOA concentration is higher with increasing catalyst loading. To further investigate the correlation between catalyst loading and TEOA concentration, the graph of optimum TEOA concentration against catalyst loading was plotted in Figure S4a at three different salinity levels. A positive and linear relationship between optimum TEOA concentration and catalyst loading regardless of salinity is observed. From the plot, an extra 3.6 vol% of TEOA is needed for every 1 g/L increment in catalyst loading to regulate the optimum condition. In the HER mechanism, TEOA must be adsorbed onto the photocatalyst; hence, a greater amount of photocatalyst requires a greater amount of TEOA for a higher reaction rate. 

**Figure 4:**
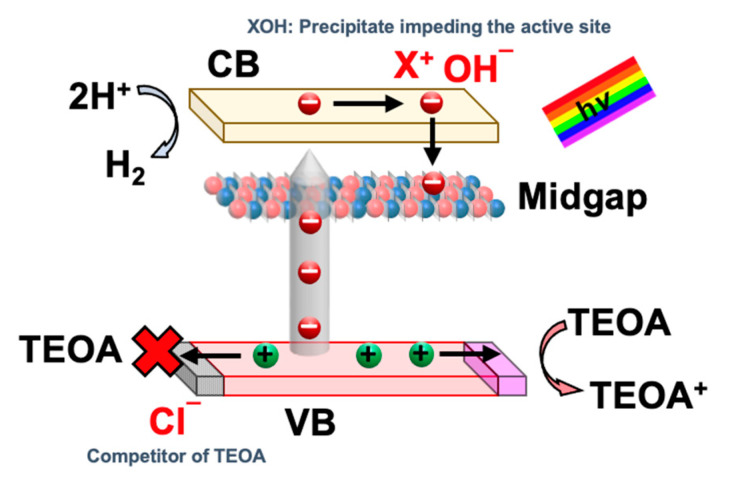
A plausible mechanism of sea salt ions towards HER from seawater splitting using ACN.

The effect of salinity on the HER activity is depicted in Figure 3c,e. Interestingly, the presence of sea salt boosts H_2_ production as compared to pure water splitting. In this work, the HER rate increases from 3.95 to 6.15 µmol/h with the uptrend of salinity from 0 to 40.2 g/L with 10 vol% TEOA and 1.625 g/L of ACN concentration. This is because the presence of Na^+^ ions adsorbed on the photocatalyst surface can promote the adsorption of a sacrificial reagent such as TEOA [16]. In this regard, TEOA can easily combine with the photogenerated holes from ACN, suppressing the charge recombination and facilitating the forward reaction [28]. While with excessive sea salt (X_3_ > 40.2 g/L), the deterioration in HER rate is observed. The decline in the HER rate could be corresponded to the undesired precipitation of insoluble hydroxide salt on the photocatalyst surface, thus hindering the reaction site [28]. In short, the adverse effects of having excessive sea salt could outweigh the positive impact of Na^+^ and hence deteriorate the HER activity. The plausible mechanism of sea salt ions towards HER from seawater splitting using ACN is postulated in Figure 4. Under low salinity conditions, the presence of Na^+^ could potentially promote the adsorption of sacrificial reagent on the surface of the photocatalyst. However, due to electrostatic attraction, cations from sea salt will be attracted to the electron-rich Pt and photocatalyst surface. Hydroxide ions (O.H.^-^), the by-product of the reduction of H_2_O molecules, exist on the Pt and photocatalyst surface and could form an ionic bond with the sea salt cations and precipitate out as insoluble salts such as Mg(OH)_2_. These insoluble salts hinder the further adsorption of H_2_O molecules onto the active site, thus deteriorating H_2_ production. Thus, the negative effect of cations overrides the positive impact of Na^+^ under high salinity. This agrees with other reported carbon nitride-based photocatalytic seawater splitting systems [29,30,31].

The optimum salinity increases as the catalyst loading increases, as shown by the contour plot in Figure 3d. To broaden the understanding of the correlation between catalyst loading and salinity, the graph in Figure S4b was plotted to show the optimum salinity with different catalyst loading for three different levels of TEOA concentration (−1, 0, +1). From the plot, a corresponding 6.3 g/L of sea salt addition is required for every gram of photocatalyst added per liter solution to maintain the optimum conditions. This positive correlation between catalyst loading and salinity is due to the greater proportion of photocatalyst, which requires a corresponded increase in Na^+^ ion to facilitate the adsorption of TEOA. 

In Figure 3f, the contour plot shows an almost negligible interaction between TEOA concentration and salinity. This agrees with the ANOVA results where the X_2_X_3_ term is insignificant due to the large *p*-value of 0.975. The negligible relationship between TEOA concentration and salinity can be further evidenced by the plot in Figure S4c, which shows a negligible slope. This indicates that the optimum salinity is not a function of the TEOA concentration.

### 3.5. Optimization and the Stability of ACN in HER from Seawater Splitting

**Figure 5:**
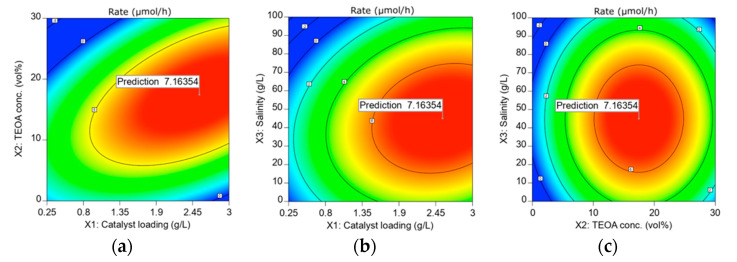
Contour plots of the interactions between (**a**) catalyst loading and TEOA concentration; (**b**) catalyst loading and salinity; (**c**) TEOA concentration and salinity at a designated range based on the HER rate.

**Figure 6:**
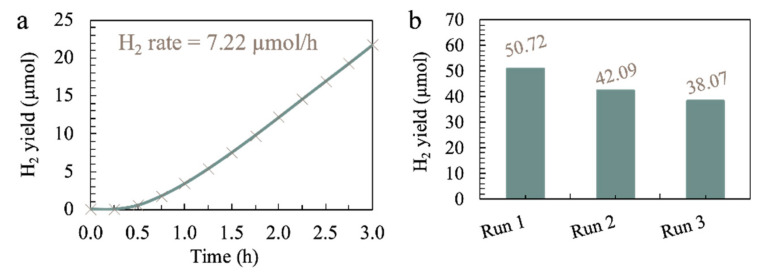
(**a**) Cumulative photocatalytic hydrogen yield over 3 h duration and (**b**) recycling runs of photocatalytic H_2_ evolution for ACN under the optimum conditions with 3 wt% Pt under simulated sunlight irradiation for 6 h each cycle.

In this study, the three factors were optimized numerically via the same software (Design Expert). The range for the factors was chosen as tabulated in Table 5, and the relevant responses are depicted in Figure 5. From the results in Figure 5, the highest HER rate of 7.16 µmol/h can be achieved when 2.55 g/L of ACN photocatalyst is employed in an aqueous 45.06 g/L sea salt solution containing 17.46 vol% TEOA as a sacrificial reagent. The HER was carried out under optimum conditions to verify the HER rate predicted by R.S.M., as depicted in Figure 6a. The experimental HER rate of 7.22 µmol/h is close to the value predicted by R.S.M. with an error of 0.84%. The photocatalytic stability of ACN was also tested by repeating the photocatalytic reaction under optimum conditions for three successive cycles, and the result is shown in Figure 6b and Figure S5. In this context, ACN maintained a reactivity of 75.1% for a total reaction time of up to 18 h. The slight reduction in H_2_ yield could be ascribed to the precipitation of insoluble salts after prolonged hours of photocatalytic reaction. Even so, ACN still renders a considerable high photocatalytic stability. 

## 4. Conclusions

The HER rate from artificial seawater splitting was well predicted by the second-order polynomial regressions calculated statistically by the Box–Behnken design method as supported by the R^2^ value of 0.9673. According to the 3D surface plots, all three factors could negatively affect the HER activity when excessive. Catalyst loading increases the HER rate by providing a greater surface site but could also lead to light-shielding when it is in excess. Subsequently, a higher TEOA concentration improves the HER rate by enhancing its adsorption rate onto the ACN. This effect could also adversely affect the HER rate because of the corresponding lower adsorption rate of water onto the photocatalyst. Furthermore, the sea salt ions (Na^+^) could favorably improve the HER rate by promoting the adsorption of TEOA and facilitating charge separation. At the same time, the excessive sea salt ions can also adversely affect the HER rate due to the reduction in surface-active sites resulting from the undesired precipitation of insoluble hydroxide salt. In addition, the R.S.M. statistical study with Box–the Behnken design shows that the single effect of X_1_, X_2_, and X_3_ are significant through the *p*-value of single and quadratic terms. The effect between X_1_ and X_2_ is significant for the interaction between the factors. However, the effect between X_1_ and X_3_ is less critical, and the effect between X_2_ and X_3_ is almost negligible. Based on the numerical optimization, the maximum HER rate is 7.16 µmol/h in the process conditions of 2.55 g/L ACN, 17.46 vol% of TEOA, and 45.06 g sea salt/L solution. The HER rate in the optimum conditions was verified experimentally, and a rate of 7.22 µmol/h was recorded (0.84% error). Lastly, ACN portrays a reasonable photostability of 75.1% over an 18 h duration under the optimum conditions.

## Figures and Tables

**Table 1:** The actual and coded values of the factors selected in Box–Behnken design.

Factors		Coded and Actual Values		
		−1	0	+1
X_1_	Catalyst loading (g/L)	0.25	1.625	3
X_2_	TEOA concentration (vol%)	0.625	10	20
X_3_	Salinity (g/L)	0	33.33	66.67

**Table 2:** Fit summary with calculated Whitcomb Scores 1 and 2 for the response—HER rate.

Source	Sequential *p*-Value	Lack of Fit *p*-Value	M	L	Predicted R^2^	Adjusted R^2^	Score 1	Score 2
Linear	0.1805	0.3327	0.2770	1	−0.0777	0.1688	−0.0215	0.0468
2FI	0.3135	0.3402	0.1595	1	0.0675	0.2494	0.0108	0.0398
Quadratic	0.0032	0.8103	1	1	0.6753	0.9083	0.6753	0.9083
Cubic	-	1	0	1	-	-	-	-

M = sequential model sum of squares score and L = lack of fit score.

**Table 3:** Design matrix, experimental determined and predicted responses for the HER in seawater via ACN photocatalyst and TEOA as a sacrificial reagent.

Run	Factors						Response	
	Catalyst Loading, X_1_ (g/L)		TEOA Conc., X_2_ (vol%)		Salinity, X_3_ (g/L)		HER Rate, Y (µmol/h)	
	Coded	Actual	Coded	Actual	Coded	Actual	Experimental	Predicted
1	−1	0.25	−1	0.625	0	33.33	1.74	1.62
2	0	1.625	−1	0.625	+1	66.67	1.31	1.64
3	0	1.625	−1	0.625	−1	0.00	negligible	0.40
4	+1	3	0	10	−1	0.00	1.99	2.20
5	0	1.625	+1	20	−1	0.00	3.87	3.54
6	0	1.625	0	10	0	33.33	5.19	6.09
7	+1	3	0	10	+1	66.67	4.43	4.71
8	+1	3	+1	20	0	33.34	6.60	6.73
9	0	1.625	0	10	0	33.34	6.42	6.09
10	0	1.625	+1	20	+1	66.67	5.23	4.82
11	+1	3	−1	0.625	0	33.33	negligible	0
12	−1	0.25	0	10	+1	66.67	1.85	1.65
13	0	1.625	0	10	0	33.33	6.65	6.09
14	−1	0.25	0	10	−1	0.00	1.93	1.64
15	−1	0.25	+1	20	0	33.33	negligible	0.60

**Table 4:** Variance analysis of second-order polynomial regressions fitted in the calculation of HER rate via Box–Behnken design.

Source	Sum of Squares	DoF	Mean Square	F-Value	*p*-Value
**Model**	82.37	9	9.15	16.42	0.0033
X_1_—Catalyst loading	6.54	1	6.54	11.74	0.0187
X_2_—TEOA conc.	23.12	1	23.12	41.48	0.0013
X_3_—Salinity	3.17	1	3.17	5.68	0.0628
X_1_X_2_	17.51	1	17.51	31.42	0.0025
X_1_X_3_	1.58	1	1.58	2.83	0.1532
X_2_X_3_	0.0006	1	0.0006	0.0011	0.9750
X_1_^2^	15.19	1	15.19	27.25	0.0034
X_2_^2^	15.15	1	15.15	27.18	0.0034
X_3_^2^	8.42	1	8.42	15.1	0.0116
**Residual**	2.79	5	0.5575		
Lack of Fit	1.72	4	0.4294	0.4015	0.8103
Pure Error	1.07	1	1.07		
**Cor Total**	85.15	14			

**Table 5:** The optimum conditions for HER via numerical optimization.

	Lower Boundary	Upper Boundary	Optimum	
Catalyst loading, X_1_	0.25	3	2.55	g/L
TEOA concentration, X_2_	0	30	17.46	vol%
Salinity, X_3_	0	100	45.06	g/L
HER Rate, Y			7.16	µmol/h

## Data Availability

Not applicable.

## Supplementary Materials

The following supporting information can be downloaded at: https://www.mdpi.com/article/10.3390/ma15144894/s1, Table S1: Composition of sea salt; Figure S1: The photoluminescence spectra of GCN and ACN; Figure S2: Field Emission Scanning Electron Microscopy of GCN. Figure S3: The photocatalytic H_2_ production rate over 6 hours duration by GCN and ACN in (a) simulated seawater under optimum conditions calculated from RSM (2.55 g/L catalyst loading, 17.46 vol% TEOA and 45.06 g/L salinity); (b) pure water (0.75 g/L catalyst loading, 10 vol% TEOA and 0 g/L salinity); Figure S4: Plots of (a) optimum TEOA concentration against catalyst loading at 3 levels of sea salt content (−1, 0, +1); (b) optimum sea salt content against catalyst loading at 3 levels of TEOA concentration (+1, 0, −1); (c) optimum sea salt content against TEOA concentration at 3 levels of catalyst loading (+1, 0, −1); Figure S5: H_2_ yield rate in 6 hours for 3 consecutive runs (Run 1−3) under optimum conditions (2.55 g/L catalyst loading, 17.46 vol% TEOA and 45.06 g/L salinity).
