# Supplementary material for: Photocatalytic Hydrogen Evolution from Artificial Seawater Splitting over Amorphous Carbon Nitride: Optimization and Process Parameters Study via Response Surface Modeling
Source: Materials (Basel). 2022 Jul 14;15(14):4894. doi: 10.3390/ma15144894 (PMC9316301; doi:10.3390/ma15144894)
Supplement: Supplementary file 1 [file materials-15-04894-s001.zip › materials-1780135-supplementary.pdf]

Supplementary Information

Photocatalytic Hydrogen Evolution via Artificial Seawater Splitting over Amorphous Carbon Nitride: Optimization and Process Parameters Study via Response Surface Modelling

Michell K. T. Chee[a], Boon-Junn Ng[a], Yi-Hao Chew[a], Wei Sea Chang[b], Siang-Piao Chai[a,*]

[a]Multidisciplinary Platform of Advanced Engineering, Chemical Engineering Discipline, School of Engineering, Monash University, Jalan Lagoon Selatan, Bandar Sunway, 47500 Selangor, Malaysia.

[b]Mechanical Engineering Discipline, School of Engineering, Monash University, Jalan Lagoon Selatan, Bandar Sunway, 47500 Selangor, Malaysia.

*Correspondence to: chai.siang.piao@monash.edu

S1 Composition of sea salt

Table S1. Composition of sea salt.

Ion	Conc.		Ion		Conc.	
Chloride	19230	mg/L	Silicon	<	0.05	mg/L
Sodium	10610	mg/L	Iron		0.012	mg/L
Sulfate	2485	mg/L	Copper		0.0003	mg/L
Magnesium	1390	mg/L	Nickel	<	0.015	mg/L
Potassium	398	mg/L	Zinc		0.008	mg/L
Calcium	409	mg/L	Manganese		0.01	mg/L
Carbonate/Bicarbonate	179	mg/L	Molybdenum		0.01	mg/L
Strontium	10	mg/L	Cobalt		0.0004	mg/L
Boron	7.6	mg/L	Vanadium	<	0.015	mg/L
Bromide	20	mg/L	Selenium	<	0.019	mg/L
Iodide	0.05	mg/L	Rubidium		0.115	mg/L
Lithium	0.26	mg/L	Barium	<	0.05	mg/L

S2 Photoluminescence spectra of GCN and GCN

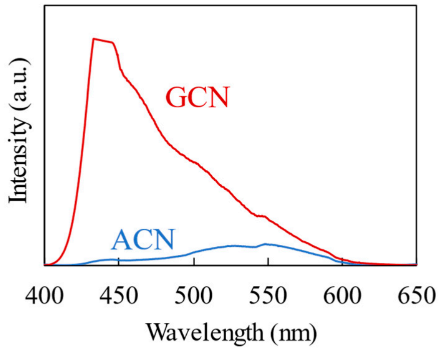

Figure S1. The photoluminescence spectra of GCN and ACN.

S3 Field Emission Scanning Electron Microscopy of GCN

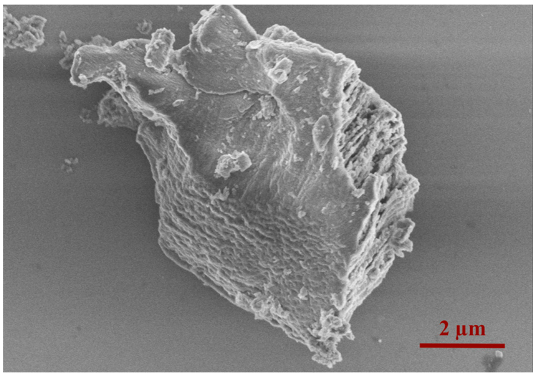

Figure S2. Field Emission Scanning Electron Microscopy of GCN.

S4 Photocatalytic performance of GCN and ACN

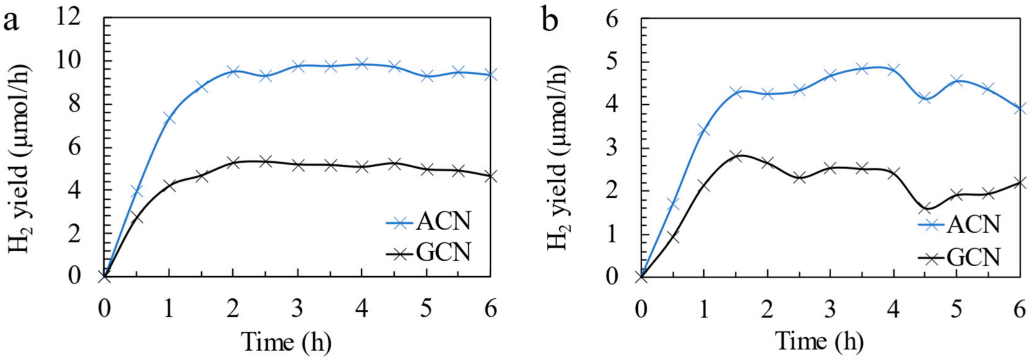

Figure S3. The photocatalytic H_2 production rate over 6 hours duration by GCN and ACN in (a) simulated seawater under optimum conditions calculated from RSM (2.55 g/L catalyst loading, 17.46 vol% TEOA and 45.06 g/L salinity); (b) pure water (0.75 g/L catalyst loading, 10 vol% TEOA and 0 g/L salinity).

S5 Plots of optimum conditions at different factorial level

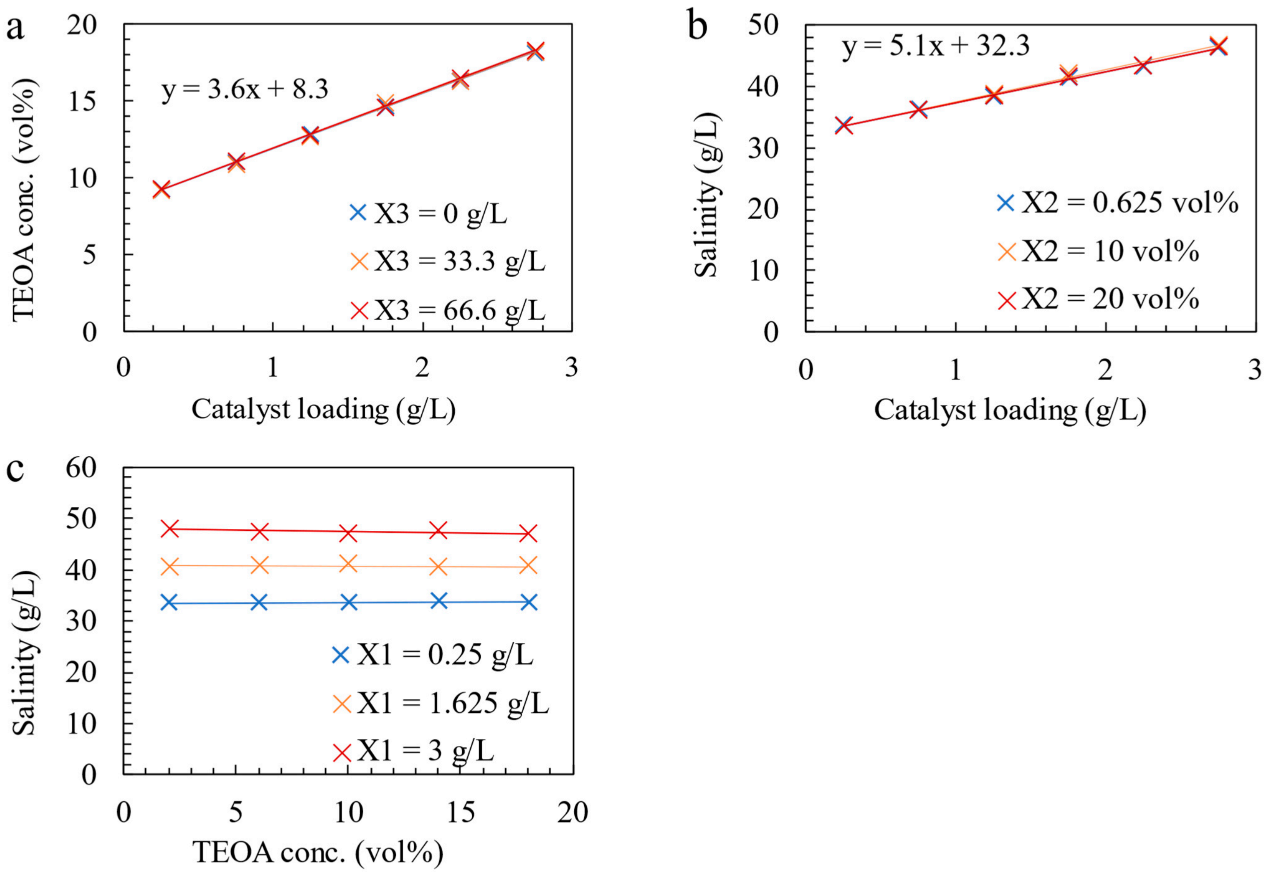

Figure S4. Plots of (a) optimum TEOA concentration against catalyst loading at 3 levels of sea salt content (−1, 0, +1); (b) optimum sea salt content against catalyst loading at 3 levels of TEOA concentration (+1, 0, −1); (c) optimum sea salt content against TEOA concentration at 3 levels of catalyst loading (+1, 0, −1).

S6 Stability test of ACN in artificial seawater under optimum conditions

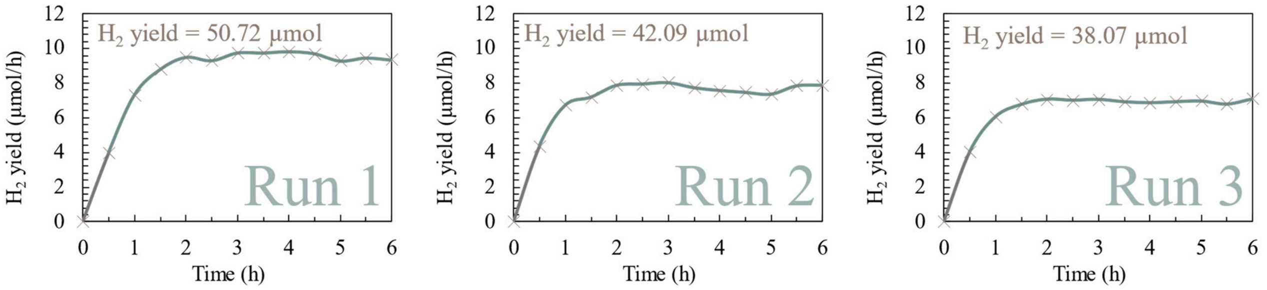

Figure S5. H_2 yield rate in 6 hours for 3 consecutive runs (Run 1−3) under optimum conditions (2.55 g/L catalyst loading, 17.46 vol% TEOA and 45.06 g/L salinity).
